# The Risk of Breast Implant-Associated Anaplastic Large Cell Lymphoma; A Systematic Review and Meta-Analysis

**DOI:** 10.1007/s00266-024-03956-9

**Published:** 2024-05-09

**Authors:** Ali Mohamed Elameen, Mohamed Awad AlMarakby, Tarek Ibrahim Atta, Asmaa Ali Dahy

**Affiliations:** 1 Department of Plastic and Reconstructive Surgery, El-Sahel Teaching Hospital, Cairo, Egypt; 2 Department of Plastic and Reconstructive Surgery, Faculty of Medicine For Girls, Al-Azhar University, Gameat Al Azhar, Nasr City, Cairo, Egypt; 3 Department of Plastic and Reconstructive Surgery, EL Helmeya Military Hospital, Military Medical Academy, Cairo, Egypt

**Keywords:** BIA-ALCL, Anaplastic large cell lymphoma, Breast implants

## Abstract

**Background:**

Breast implant-associated anaplastic large cell lymphoma (BIA-ALCL) is an emerging disorder that has gained global attention throughout the past era. The present meta-analysis was performed to retrieve the risk of BIA-ALCL from population-based epidemiological studies. Factors associated with BIA-ALCL were evaluated to identify patients at higher risk of BIA-ALCL.

**Methods:**

A systematic literature search was executed throughout 12 databases. All epidemiological studies encompassing patients with breast implants either for aesthetic or reconstructive purposes and reported the risk of BIA-ALCL were included. Studies reported the risk factors of BIA-ALCL were included.

**Results:**

The present meta-analysis included 17 articles, encompassing 525,475 patients with breast implants. There were 254 patients with BIA-ALCL with a mean duration to the diagnosis of BIA-ALCL of 13.16 years (95% CI 11.7–14.6, *P* < 0.001). There were 44 patients with textured breast implants and two with smooth implants. Patients with breast implants were 28.86 times more at high risk of BI-ALCL (95% CI 3.123–266.681). The risk ranged from 0 to 1 per 1000 cases with breast implants, with a similar risk among patients seeking aesthetic and reconstructive surgeries. The risk was 0 to 1 case per 1000 cases among patients with textured breast implants. There was a significant association between the history of breast cancer and BIA-ALCL (*P* = 0.0016).

**Conclusion:**

This meta-analysis confirmed the association between breast implants and ALCL. There was a similar risk of BIA-ALCL among patients with aesthetic or reconstructive surgeries. Patients with a history of breast cancer were at higher risk of BIA-ALCL.

**Level of Evidence III:**

This journal requires that authors assign a level of evidence to each article. For a full description of these Evidence-Based Medicine ratings, please refer to the Table of Contents or the online Instructions to Authors www.springer.com/00266.

**Supplementary Information:**

The online version contains supplementary material available at 10.1007/s00266-024-03956-9.

## Introduction

Aesthetic and reconstructive breast surgeries are among the most performed surgical procedures worldwide. In the USA, more than 500,000 patients are undergoing aesthetic breast surgeries annually, in which augmentation mammoplasty is the most common procedure [1, 2]. The more patients are diagnosed with breast cancer, the more women seek breast reconstruction surgeries. Implant-based breast reconstruction (IBBR) is the most carried out reconstructive surgery after mastectomy. IBBR accounts for more than 80% of all women subjecting to breast reconstruction [3]. Worldwide, more than 35 million women are subjected to implant-based breast surgeries, with nearly 450 thousand breast implants used yearly in the USA [4, 5]. The increasing demand for implant-based breast surgeries reflects the ultimate need for women to recreate aesthetically pleasant breasts with acceptable functional and physical outcomes [6].

The advancement in implant manufacturing techniques considerably improved the outcomes of breast surgeries. Implants maintain the anatomical boundaries of the breasts, allowing for more aesthetically appealing natural breast [7]. Breast implants are categorized based on surface roughness, filling materials, and shape. Based on the surface of the implants, these devices are assorted into textured or smooth implants. Recently, there has been an increased usage rate of smooth breast implants at the expense of textured breast implants [8]. This might be attributed to the considerable degree of biofilm formation and seroma associated with textured breast implants [9]. The biofilm surrounding the textured implants may stimulate lymphocyte production, which triggers a cycle of inflammation that ultimately results in breast implant-associated anaplastic large cell lymphoma (BIA-ALCL) [10].

BIA-ALCL has raised concerns about the safety and future of breast implants. It is a rare type of T-Cell lymphoma that arises around the breast implants. It is the disease of the breast implant capsule and not of the breast tissue itself [11]***.*** However, lack of knowledge is a significant barrier to managing patients with BIA-ALCL appropriately, with little being known regarding the relationship between breast implants and ALCL. This results in considerable debate about the future safety of breast implants in aesthetic and reconstructive settings [12].

The growing interest in BIA‐ALCL reflects its implications for implant-based breast surgeries. A possible correlation between breast implants and ALCL could change the reconstructive options, choice of breast implants, and the future of other implanted devices [13]. The exact number of patients with BIA-ALCL is challenging to determine due to the lack of global disease awareness [14]. Despite the progress in calculating the risk of BIA-ALCL, the results of previously published systematic reviews are inconsistent [15, 16]. There is a considerable variation in the estimated current risk of BIA-ALCL. This variation may be due to underreporting, duplicated cases, misdiagnosis, difficulty obtaining a valid clinical history from the affected cases, differences in the diagnostic approaches, and inadequate data related to the total number of patients with breast implants [17].

A comprehensive understanding of breast implant safety remains a common goal of plastic and reconstructive surgeons [18]. Herein, the current meta-analysis was performed to retrieve the risk of BIA-ALCL from population-based epidemiological studies. This risk was categorized based on the type of breast implant and breast surgery. Furthermore, factors associated with BIA-ALCL were evaluated to identify patients at higher risk of BIA-ALCL. Recognizing such evidence is crucial to ensuring the safety of implant-based breast surgeries and optimizing preventive measures for patients at higher risk of BIA-ALCL.

## Materials and Methods

The Preferred Reporting Items for Systematic Reviews and Meta-Analysis (PRISMA) guidelines [19] and the recommendations of the Cochrane Collaboration [20] were followed while conducting the present study. This systematic review was reported based on the guidelines of Meta-analysis of observational studies in epidemiology [21] (Supplementary Table 1).

### Study Selection

All epidemiological studies encompassed patients with breast implants either for aesthetic or reconstructive purposes and reported the number of patients who developed BIA-ALCL apart from the total cohort were included. Studies reported the risk factors of BIA-ALCL in patients with breast implants were included. There was no restriction on the patient’s age, sex, race, or place. On the contrary, non-epidemiology-based studies or those that did not report the associations between breast implants and anaplastic large cell lymphoma (ALCL) were ousted. Studies with unextractable data, guidelines, review articles, case reports, non-human studies, case series, comments, editorials, posters, letters, and books were excluded. The screening processes were performed blindly to retrieve the possibly relevant articles. The discrepancy between the reviewers was solved by discussion. The screening and eligibility processes were reported using PRISMA flow chart.

### Data Source

A comprehensive systematic literature review was carried out throughout twelve databases from inception until 16 April 2023. The following databases were searched: Google Scholar, PubMed, Web of Science (ISI), SIGLE, Scopus, Virtual Health Library (VHL), Clinical trials, Controlled Trials (RCT), NYAM, Cochrane Collaboration, EMBASE, and WHO International Clinical Trials Registry Platform (ICTRP). No restrictions were employed on patients’ age, ethnicity, race, language, or place. The search strategy applied individualized controlled vocabulary terms based on each searched database’s guidelines. A combination of medical subject headings and text words was used to retrieve a wide range of potentially eligible articles. Citation tracking, cross-referencing, and screening of all references of previously published systematic reviews were performed. The following keywords were used in every possible combination; ‘Breast’, ‘Mammary’, ‘Implant’, ‘Implants’, ‘Prosthesis’, ‘Expander’, ‘BIA-ALCL’, ‘Anaplastic’,’ Lymphoma,’ ‘Lymphomas’, ‘lymphoproliferative’.

### Data Extraction

The demographic characteristics of the included studies were retrieved. This included study’s title, the first author’s second name, study design, year of publication, study period, and study region. Furthermore, patients’ demographic characteristics were extracted, including the total number of patients with breast implants, the number of patients who developed BIA-ALCL, patients’ age at the diagnosis of BIA-ALCL, diagnostic workup, and history of breast cancer. The data related to breast implants were extracted, including the reasons for breast implants, time from implants to the diagnosis of BIA-ALCL, times of breast implants, types of breast implants, side of the implants, and the number of implants. The data related to BIA-ALCL were retrieved, including the lymphoma localization, type of ALCL, stage of BIA-ALCL, and treatment-related data. The data were extracted independently in a well-structured Microsoft Excel spreadsheet.

### Quality Assessment of the Included Studies

The quality of the observational studies was calculated using the National Institute of Health (NIH) quality assessment tool [22]. The quality of the included studies was assorted into good, fair, and bad when the score was > 65%, 30–65%, and < 30%, respectively.

### Statistical Analysis

The pooled summary of odds ratio (OR) was computed by pooling the effect sizes from all the included articles to confirm the association between breast implants and ALCL. The cumulative incidence of BIA-ALCL was estimated by pooling the incidence value and their 95% CI (confidence intervals) to calculate the pooled cumulative incidence. The prevalence of BIA-ALCL was calculated by calculating the event rate and 95% CIs for each study and followed by pooling the effect sizes of all studies to evaluate the summary risk with 95% CI. The fixed-effect model was used when a fixed population effect size was assumed. Conversely, the random-effects model was implemented when the statistical heterogeneity was established. Statistical heterogeneity was calculated using Higgins I^2^ statistic, and the Cochrane Q (*χ*^2^ test), at the value of > 50% and *P* < 0.10, respectively [23]. Publication bias was revealed with the asymmetrical distribution of the included studies within the funnel plot along the middle line and based on Egger’s regression test (*P*-value < 0.10). The trim and fill method of Duvall and Tweedie was executed to investigate the impact of publication bias on the overall significance level [24].

Random-effects meta-regression was conducted to explore the potential associations with BIA-ALCL. Subgroup analysis was performed based on the type of breast surgery, the types of breast implants, and based on study regions. The data were analysed using Comprehensive Meta-Analysis v3 software. The significance was revealed at the value of *P *< 0.05 [25].

## Results

**Fig. 1:**
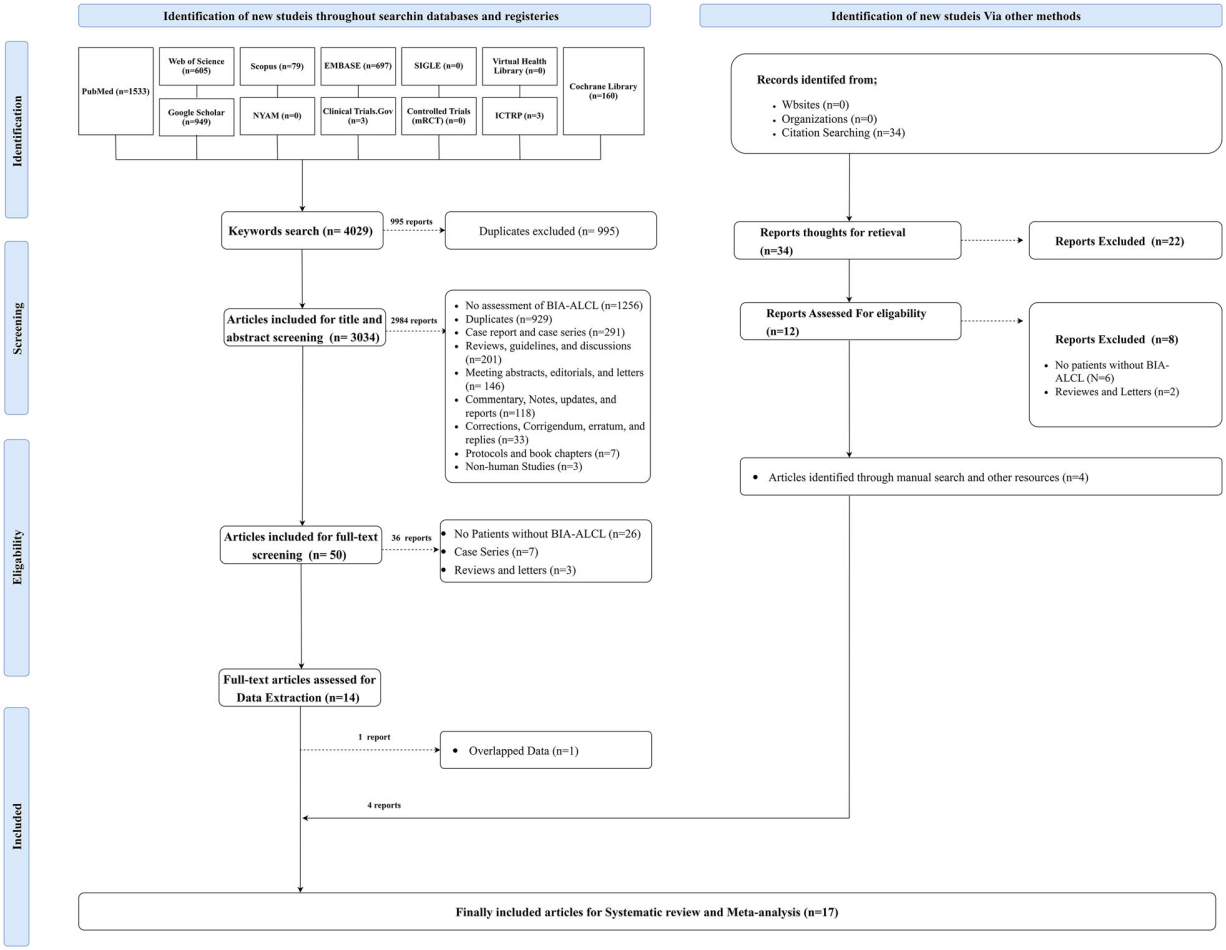
PRISMA 2020 flow diagram for updated systematic reviews which included searches of databases, registers, other sources, and screening

A systematic literature search of 12 databases revealed 4029 articles. Of them, 995 reports were excluded as duplicates, resulting in 3034 articles eligible for screening. The title and abstract screening process excluded 2984 studies, yielding 50 articles suitable for full-text screening. Consequently, 36 studies were excluded, resulting in 14 articles included for data extraction, in which one study was excluded. Four studies were identified through manual searching, resulting in 17 articles that were finally included for systematic review and meta-analysis. The literature search process, identification of studies, and eligibility are shown in Fig. 1

### Demographic Characteristics and Quality of the Included Studies

The present systematic review and meta-analysis included 17 articles, encompassing 525475 patients with breast implants [26–42]. There were ten articles of prospective study design and seven reports of retrospective design. Ten articles included data from the USA. There were 254 patients with BIA-ALCL throughout the follow-up periods, which ranged from five to 30.9 years. The average age at diagnosis of BIA-ALCL ranged from 24 to 77 years, with an average time from implants to diagnosis ranging from one to 39 years with a mean duration of 13.16 years (95% CI 11.7–14.6, *P* < 0.001). There were 42 patients with a history of breast cancer (Table 1).

**Table 1:** Demographic characteristics of the included studies with patients with BIA-ALCL

Study ID		Study region	Study design	Registration number	Study period	BIA-ALCL Cases	Total cohort	Age at diagnosis
						Number	Number	Median/range
1	Adam et al. [26]	Sweden	Prospective cohort	NA	NR	0	42,035	NR
2	Boer et al. [29]	Netherlands	Population-based case-control	NA	1990 and 2016	32	3000	59 (24–87)
3	Cordeiro et al. [27]	USA	Prospective cohort study	NA	December 1992 to December 2017	10	3546	60 (53–73)
4	Coroneos et al. [28]	USA	Prospective cohort	NA	February 2007 to March 2010	1	99993	NR
5	Kinslow et al. [30]	USA	Retrospective	NA	2000 to 2018	5	56784	50–54 (40–59)
6	Largent et al. [31]	France	Retrospective	NA	NR	3	89382	59.33 ± 9.39*
7	Maxwell et al. [35]	USA	Prospective, multicenter	NCT00690339	February 2001 and February 2002	1	941	NR
8	McGuire et al. [36]	USA	Retrospective	NA	NR	4	17655	NR
9	Nelson et al. [37]	USA	Retrospective	NA	July 1, 1991 and June 30, 2017	11	9373	54.57 (50.39–77.33)
10	Nelson et al. [38]	USA	Retrospective cohort	NA	1995-2016	0	3310	NR
11	Singh et al. [39]	USA	Prospective cohort	NCT00443274	March of 2010 to October 1, 2015	0	55279	NR
12	Spear et al. [40]	USA	Prospective cohort	NA	January of 1999 and June of 2000	0	715	NR
13	Vase et al. [41]	Denmark	Prospective cohort	NA	1973–2010	0	19,885	NR
14	Wang et al. [42]	USA	Prospective cohort	NA	1 January 1995 and 31 December 2012,	2	123392	NR
15	Loch-Wilkinson et al. [32]	Australia and New Zealand	Prospective cohort	NA	2007 and 2016	55	NR	NR
16	Loch-Wilkinson et al. [33]	Australia	Prospective cohort	NA	October 2015 to May 2019	104	NR	NR
17	Magnusson et al. [34]	Australia and New Zealand	Prospective cohort	NA	January of 2017 and April of 2018	26	NR	NR

Study ID		Reasons for breast implants		Type of reconstruction		History of breast cancer	Previous therapies	
		Aesthetic	Reconstruction	Unilateral	Bilateral		Radiotherapy	Chemotherapy
		Number	Number	Number	Number	Number	Number	Number
1	Adam et al. [26]	0	0	NR	NR	NR	0	0
2	Boer et al. [29]	22	10	NR	NR	10	NR	NR
3	Cordeiro et al. [27]	0	10	NR	NR	10	NR	NR
4	Coroneos et al. [28]	NR	NR	NR	NR	NR	NR	NR
5	Kinslow et al. [30]	0	5	NR	NR	5	NR	NR
6	Largent et al. [31]	0	3	3	NR	3	1	2
7	Maxwell et al. [35]	0	1	NR	NR	1	0	0
8	McGuire et al. [36]	2	2	NR	NR	2	NR	NR
9	Nelson et al. [37]	0	11	1	10	11	NR	NR
10	Nelson et al. [38]	0	0	NR	NR	0	0	0
11	Singh et al. [39]	0	0	NR	NR	0	0	0
12	Spear et al. [40]	0	0	NR	NR	0	0	0
13	Vase et al. [41]	0	0	NR	NR	0	0	0
14	Wang et al. [42]	NR	NR	NR	NR	NR	NR	NR
15	Loch-Wilkinson et al. [32]	NR	NR	NR	NR	NR	NR	NR
16	Loch-Wilkinson et al. [33]	NR	NR	NR	NR	NR	NR	NR
17	Magnusson et al. [34]	NR	NR	NR	NR	NR	NR	NR

*BIA-ALCL* breast implant associated anaplastic large cell lymphoma, *NA* non applicable, *NR* non-reported

*Data reported using mean and standard deviation

There were 44 patients with textured breast implants, and two with smooth implants had BIA-ALCL. Furthermore, there were 28 patients with stage I BIA-ALCL and six with stage II BIA-ALCL. Thirty-three patients received surgical therapy; 15 patients were treated with chemotherapy in addition to the surgical treatment. The included observational studies revealed a fair quality of four studies [26, 28, 36, 40]. The quality of the remaining 13 articles was good (Table 2).

**Table 2:** Characteristics of patients with BIA-ALCL and quality assessment of the included studies

Study ID		Time to BIA-ALCL diagnosis	Types of implants		Filling material		Stage of BIA-ALCL	
			Textured	Smooth	Silicone	Saline	I	II
			Number	Number	Number	Number	Number	Number
1	Adam et al. [26]	NR	0	0	0	0	NR	NR
2	Boer et al. [29]	13 (1–39)	22	0	32	0	21	5
3	Cordeiro et al. [27]	11.7 (7.4–15.8)	10	0	5	5	7	1
4	Coroneos et al. [28]	NR	NR	NR	NR	NR	NR	NR
5	Kinslow et al. [30]	NR	NR	NR	NR	NR	NR	NR
6	Largent et al. [31]	NR	2	1	1	NR	NR	NR
7	Maxwell et al. [35]	NR	NR	NR	NR	NR	NR	NR
8	McGuire et al. [36]	3.5–11.6	NR	NR	4	0	NR	NR
9	Nelson et al. [37]	10.26 years (6.43–15.52)	10	1	NR	NR	NR	NR
10	Nelson et al. [38]	NR	0	0	0	0	NR	NR
11	Singh et al. [39]	NR	0	0	0	0	NR	NR
12	Spear et al. [40]	NR	0	0	0	NA	NR	NR
13	Vase et al. [41]	NR	0	0	0	0	NR	NR
14	Wang et al. [42]	NR	NR	NR	NR	NR	NR	NR
15	Loch-Wilkinson et al. [32]	7.46 years	NR	NR	NR	NR	NR	NR
16	Loch-Wilkinson et al. [33]	NR	NR	NR	NR	NR	NR	NR
17	Magnusson et al. [34]	NR	NR	NR	NR	NR	NR	NR

Study ID		Stage of BIA-ALCL		Treatment of BIA-ALCL			Follow-up period	Quality assessment	
		III	IV	Surgical therapy	Surgical therapy and chemotherapy	Chemotherapy and hematopoietic stem cell transplant			
		Number	Number	Number	Number	Number		%	Decision
1	Adam et al. [26]	NR	NR	NR	NR	NR	11.7 years (1 to 14)	61.5	Fair
2	Boer et al. [29]	3	3	11	12	9	33( 2–240 months)	69.23	Good
3	Cordeiro et al. [27]	2	0	9	1	NR	8.1 years (3 months–30.9 years).	69.23	Good
4	Coroneos et al. [28]	NR	NR	NR	NR	1	7 years	61.5	Fair
5	Kinslow et al. [30]	NR	NR	NR	NR	NR	81 (46–125) months	69.23	Good
6	Largent et al. [31]	NR	NR	NR	2	NR	10 years	69.23	Good
7	Maxwell et al. [35]	NR	NR	NR	NR	NR	10 years	76.92	Good
8	McGuire et al. [36]	NR	NR	NR	NR	NR	10 years	61.5	Fair
9	Nelson et al. [37]	NR	NR	NR	NR	NR	26 years	76.92	Good
10	Nelson et al. [38]	NR	NR	NR	NR	NR	6.8 years	69.23	Good
11	Singh et al. [39]	NR	NR	NR	0	NR	5 years	69.23	Good
12	Spear et al. [40]	NR	NR	NR	0	NR	10 years	61.5	Fair
13	Vase et al. [41]	NR	NR	NR	NR	NR	37 years	69.23	Good
14	Wang et al. [42]	NR	NR	NR	NR	NR	17 years	69.23	Good
15	Loch-Wilkinson et al. [32]	NR	NR	NR	NR	NR	9 years	69.23	Good
16	Loch-Wilkinson et al. [33]	NR	NR	NR	NR	NR	NR	69.23	Good
17	Magnusson et al. [34]	NR	NR	NR	NR	NR	NR	69.23	Good

*BIA-ALCL* breast implant associated anaplastic large cell lymphoma, *NA* non applicable, *NR* non-reported

### The Risk of BIA-ALCL

#### Total Risk of BIA-ALCL

**Fig. 2:**
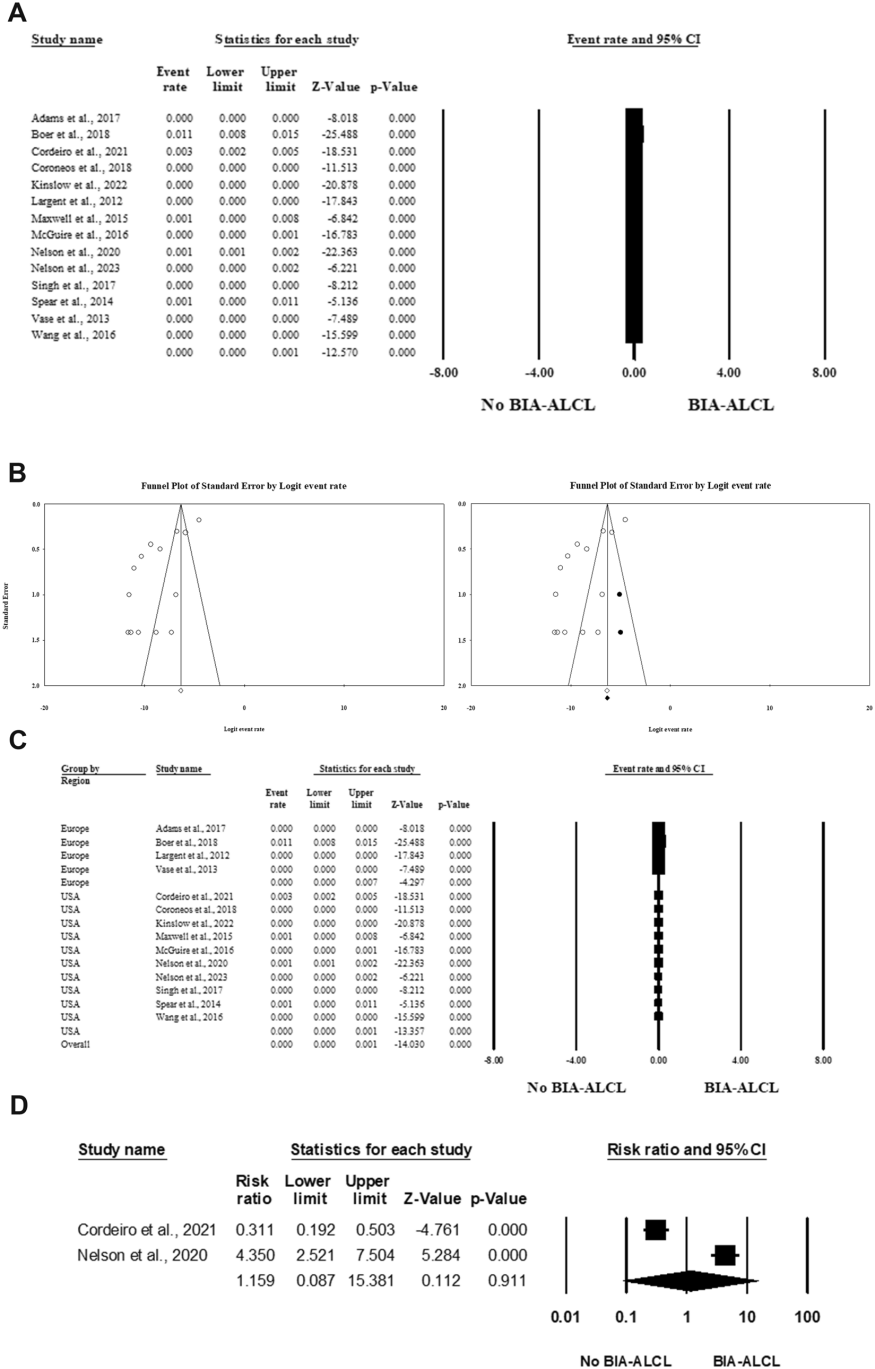
Forest plot of summary analysis of the (**A**) Event rate and 95% CI of the prevalence of BIA-ALCL among patients with breast implants. (**B**) Funnel plots showing evidence of publication bias on the left side and after imputing the studies on the right side. (**C**) Subgroup analysis of the event rate and 95% CI of the prevalence of BIA-ALCL among patients with breast implants based on the regions of the included studies. (**D**) Cumulative incidence of BIA-ALCL per 1000 patients per year among patients with breast implants. The size of the black squares is proportional to the statistical weight of each trial. The black diamond represents the pooled point estimate. The positioning of both diamonds and squares (along with 95% CIs) beyond the vertical line (unit value) suggests a significant outcome

Fourteen studies included 525475 patients with breast implants and reported the number of cases of BIA-ALCL [26–31, 35–42]**.** In the random-effects model (*I*^2^ = 96.08%, *P* < 0.001), the risk of BIA-ALCL ranged from 0 to 1 per 1000 cases of breast implants (event rate 0, 95% CI 0–0.1; *P* < 0.001). Evidence of publication bias was detected by the asymmetrical distribution of studies along the middle line of the funnel plot and based on Egger’s regression test (Intercept = − 5.43, *P* = 0.00124). The trim and fill method was applied to assess the impact of publication bias on the overall estimate after imputing possible missing studies. This revealed no significant impact of publication bias on the significant level of the overall estimate (*P* < 0.001) (Fig. 2A and B).

Subgroup analysis based on the study regions revealed a relatively high prevalence of BIA-ALCL in the European region. The prevalence of BIA-ALCL ranged from 0 to 7 cases per 1000 patients with breast implants in Europe (event rate 0, 95% CI 0–0.7; *P* < 0.001), in contrast to 0 to 1 case per 1000 patients in the USA (event rate 0, 95% CI 0–0.1; *P* < 0.001) (Fig. 2C).

#### Cumulative Risk of BIA-ALCL

Two studies included 12919 cases with breast implants that reported the cumulative incidence of BIA-ALCL per 1000 patients per year [27, 37]. In the random-effects model (*I*^2^ = 98.023%, *P* < 0.001), the cumulative risk of BIA-ALCL per 1000 persons per year was 1.159 (0.087 to 15.381, *P* = 0.911) (Fig. 2D).

#### The Risk of BIA-ALCL Based on the Type of Breast Surgery

**Fig. 3:**
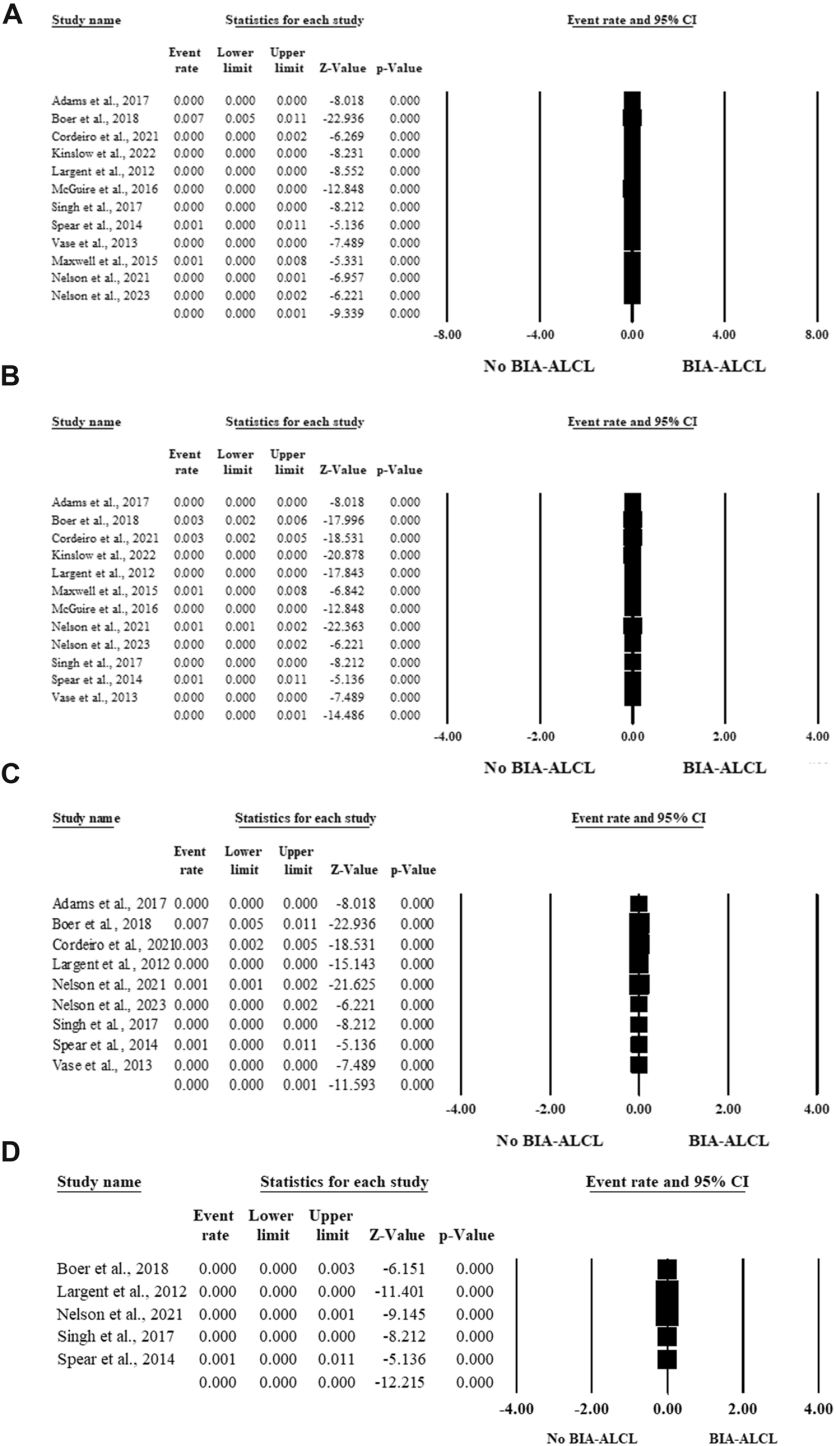
Forest plot of summary analysis of the (**A**) Event rate and 95% CI of the prevalence of BIA-ALCL among patients seeking breast implants for aesthetic purposes. (**B**) Event rate and 95% CI of the prevalence of BIA-ALCL among patients seeking breast implants for reconstructive purposes. (**C**) Event rate and 95% CI of the prevalence of BIA-ALCL among patients with textured breast implants. (**D**) Event rate and 95% CI of the prevalence of BIAALCL among patients with smooth breast implants. The size of the black squares is proportional to the statistical weight of each trial. The black diamond represents the pooled point estimate. The positioning of both diamonds and squares (along with 95% CIs) beyond the vertical line (unit value) suggests a significant outcome

The risk of BIA-ALCL among patients seeking breast implants for aesthetic purposes was evaluated among twelve articles, including 301905 patients [26, 27, 29–31, 35–41]. The prevalence of BIA-ALCL ranged from 0 to 1 case per 1000 patients with breast implants (Event rate= 0, 95% CI 0–0.1, *P* < 0.001) in the random-effects model (*I*^2^ = 91.99%, *P* < 0.001). Twelve articles included 301905 patients with breast implants for reconstructive purposes [26, 27, 29–31, 35–41]**.** In the random-effects model (*I*^2^ = 91.3%, *P* < 0.001), the prevalence of BIA-ALCL among patients with breast implants for reconstructive surgeries ranged from 0 to 1 case per 1000 patients with breast implants (Event rate= 0, 95% CI 0–0.1, *P* < 0.001) (Fig. 3A and B).

#### The Risk of BIA-ALCL Based on the Types of Breast Implants

Nine articles included 226525 patients with breast implants that reported the prevalence of BIA-ALCL among patients with textured implants [26, 27, 29, 31, 37–41]. The risk of BIA-ALCL was 0 (95% CI 0–0.001) with a risk ranging from 0 to one per 1000 cases with textured breast implants (*P*<0.001) in the random-effects model (*I*^2^ = 93.48%, *P* < 0.001). (Fig. 3C)

Five articles included 177634 patients with breast implants and reported the risk of BIA-ALCL among patients with smooth implants [29, 31, 37, 39, 40]. The risk of BIA-ALCL was 0 among patients with smooth breast implants (*P* < 0.001) in the random-effects model (I^2^ = 52.64%, *P* = 0.076) (Fig. 3D).

**Fig. 4:**
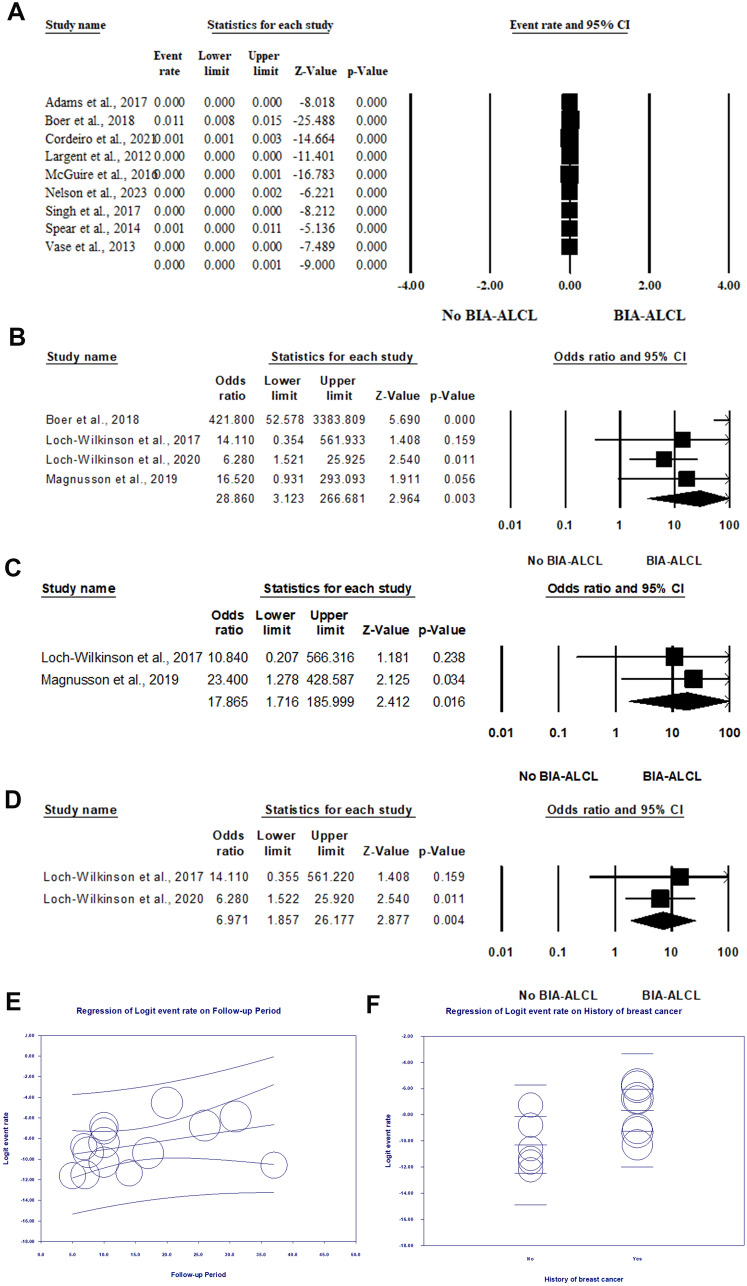
Forest plot of summary analysis of the (**A**) Event rate and 95% CI of the prevalence of BIA-ALCL among patients with silicone breast implants. (**B**) Odds ratio (OR) and 95% CI of the association between breast implants and ALCL. (**C**) Odds ratio (OR) and 95% CI of the association between polyurethane breast implants and ALCL. (**D**) Odds ratio (OR) and 95% CI of the association between biocell textured breast implants and ALCL. (**E**) Weighted random-effects meta regression analysis regressing risk of BIA-ALCL against the follow-up period. (**F**) Weighted random-effects meta regression analysis regressing risk of BIA-ALCL against the history of breast cancer. The size of the black squares is proportional to the statistical weight of each trial. The black diamond represents the pooled point estimate. The positioning of both diamonds and squares (along with 95% CIs) beyond the vertical line (unit value) suggests a significant outcome

The risk of BIA-ALCL among patients with silicone breast implants was evaluated within nine articles encompassing 234807 patients with breast implants [26, 27, 29, 31, 36, 38–41]. In the random-effects model (I^2^ = 94.97%, *P* < 0.001), the risk of BIA-ALCL ranged from 0 to one per 1000 cases (event rate 0; 95% CI 0–0.001; *P* < 0.001) (Fig. 4A).

### Factors Associated with BIA-ALCL

#### Association between types of breast implants and ALCL

Four articles reported the association between breast implants and ALCL [29, 32–34]. In the random-effects model (I^2^ = 72.28%, *P* = 0.013), patients with breast implants were 28.86 times more vulnerable to developing BIA-ALCL (OR 28.86, 95% CI 3.123–266.681, *P* = 0.003) (Fig. 4B).

Two articles [32, 34] reported the association between polyurethane breast implants and BIA-ALCL. In the random-effects model (I^2^ = 0%, *P* = 0.75), patients with polyurethane breast implants were at 17.86 times higher risk of developing BIA-ALCL (OR 17.86, 95%CI; 1.71 to 185.99, *P* = 0.016). In this respect, two studies [32, 33] evaluated the association between biocell textured breast implants and BIA-ALCL. The risk of BIA-ALCL was 6.971 times among patients with biocell textured implants (OR 6.971, 95% CI 1.857–26.177, *P* = 0.004) in the random-effects model (I^2^ = 0%, *P* = 0.688) (Fig. 4C and D).

#### Association between BIA-ALCL and Follow-up periods

Fourteen articles comprised 525290 patients with breast implants included for meta-regression analysis [26–31, 35–42]. The analysis revealed no statistically significant impact of follow-up periods on the risk of BIA-ALACL (Coefficient = 0.0902, 95% CI − 0.041, 0.22; *P* = 0.18) with proportional of the total between study variance explained by the model (R^2^) of 0.18. (Fig. 4E)

#### Association between BIA-ALCL and history of breast cancer

The association between the history of breast cancer and the risk of BIA-ALCL was evaluated within 13 articles, including 401898 patients with breast implants [26–31, 35–41]. The random meta-regression model revealed a statistically significant positive association between the history of breast cancer and BIA-ALCL (*P* = 0.0016) with a coefficient of 2.63 (95% CI 0.47, 4.97). The model explained 22% of the total between study variance with R^2^ of 0.22 (Fig. 4F).

## Discussion

BIA-ALCL is an emerging disorder that has gained global attention throughout the past era [43]. The knowledge regarding the prevalence, risk factors, and management of BIA-ALCL needs to be more extensive. The current literature is insufficient to draw conclusive evidence for current clinical practice. This is because most previously published systematic reviews are based on case reports without quantitative data synthesis [44]. Therefore, the present meta-analysis was conducted to retrieve the available evidence from well-structured epidemiological studies on the prevalence and risk factors of BIA-ALCL.

The present study confirmed the association between breast implants and ALCL. The risk of BIA-ALCL ranged from 0 to 1 per 1000 cases, with a similar risk of BIA-ALCL among patients who received breast implants for reconstruction and aesthetic purposes. The risk of BIA-ALCL was relatively low among patients with textured or smooth breast implants. Breast implants increase the risk of BIA-ALCL 28.86 times more than the risk of ALCL among the general population, with a higher risk among patients with polyurethane breast implants. Patients with a history of breast cancer were at higher risk of BIA-ALCL. These findings highlight the ultimate role of comprehensive counselling for patients seeking implant-based breast surgeries regardless of the type of surgery and type of breast implants. This counselling should include the risk and symptoms of BIA-ALCL with close follow-up for the most susceptible patients [45].

The current meta-analysis showed a relatively similar risk of BIA-ALCL between textured and smooth implants. Consistent with this finding, Nava et al., highlighted the non-exclusive association between textured implants and BIA-ALCL [46]. These findings highlighted a potential risk of publication bias in this issue as many previously published studies revealed only risk of BIA-ALCL with textured implants. Lynch et al. reported a risk of BIA-ALCL associated with textured implants ranging from 1:355 to 1:5100. However, this systematic review reported no BIA-ALCL associated with smooth breast implants [15]. Furthermore, Gidengil et al. reported most cases of BIA-ALCL were associated with textured implants. They highlighted the limited interpretation of these findings without knowledge of the total number of patients with textured implants and other types of implants in the general population [47]. The lack of accurate data about the denominator in the previously published studies conveys a high risk of bias regarding the precise risk of BIA-ALCL. The present meta-analysis included only epidemiology-based studies that identified the denominator to tackle the limitations of the previously published reviews.

The current meta-analysis revealed a relatively higher risk of BIA-ALCL in Europe than in the USA. In the USA, the use of textured implants peaked in 2016 with a utilization rate of nearly 18% and 41% for aesthetic and reconstructive breast surgeries. Based on the Dutch Breast Implant Registry, textured implants are used in 88% of cases, while smooth implants are used in 7 to 11% of aesthetic breast surgeries in Europe. The substantial variation in the risk of BIA-ALCL between the USA and Europe may reveal the role of ethnicity and genetic predisposition in developing BIA-ALCL. Furthermore, the large proportion of patients with textured implants outside the USA may be associated with the high rate of reporting cases with BIA-ALCL associated with textured implants [48, 49]. Noteworthy, most of the data from the USA is related to the FDA alert of implant safety and the significant number of implant-based breast surgeries. BIA-ALCL is extremely rare in Africa, Asia, and Latin America, with no epidemiology-based studies including patients from these regions. This reflects the risk of underreporting bias and highlights the need to establish validated registries in these regions to estimate the risk of BIA-ALCL accurately.

The present meta-analysis revealed a higher risk of BIA-ALCL associated with breast implants. This risk was approximately threefold higher among patients with polyurethane breast implants than those with textured biocell breast implants. This finding may reveal a proportionate relationship between the risk of BIA-ALCL and surface texturization. Parallel with these findings, Girardi et al., reported an association between ALCL and breast implants, with comprehensive knowledge about the disease that should be given to all patients before breast implant surgeries [50]. These findings infer a more significant association between breast implants and ALCL but not an exclusive causal relationship. There is an urgent need for more data about the etiopathogenesis of BIA-ALCL since the number of events is small owing to the rarity of the disease. The association between implant types and the risk of BIA-ALCL necessitates further evaluation and should be studied in the context of other confounders. This is because of the limited clinical data in the literature regarding the implant types, texturization, manufacturing, and the risk of BIA-ALCL [4, 51].

There was a considerable risk of BIA-ALCL among patients with a history of breast cancer. This finding demonstrates some patients’ immunological and genetic predisposition to develop BIA-ALCL. Parallel with these findings, Jabagi et al., reported a higher risk of haematological malignancies after post-operative breast cancer treatment, particularly among young patients who received radiotherapy or chemotherapy [52]. Wang et al., reported an increased risk of non-Hodgkin lymphoma after radiation therapy among patients with breast ductal carcinoma [53]. Exposure to high dosages of ionizing radiation results in chromosomal alterations and somatic mutations. This increases the risk of myeloid malignancies, leukaemias, and lymphomas, particularly in the irradiated organs [54, 55]. Alkylating chemotherapeutic agents induce mutations of the genes responsible for cell proliferation and tumour suppressors, activating the oncogenic cascade. This results in abnormal cell differentiation and proliferation, resulting in haematological malignancies [56, 57]. These findings highlighted the importance of close follow-up for patients with a history of breast cancer and subjected to implant-based breast surgeries either for reconstructive or aesthetic purposes.

The present systematic review and meta-analysis gathered the dispersed evidence related to the risk of BIA-ALCL in the literature. The study included only epidemiological studies, allowing for estimating the current risk of BIA-ALCL among the largest cohort in the literature. However, some limitations were to be considered while interpreting the retrieved evidence for the current clinical practice. Many of the included articles were retrospective designs with a considerable risk of information bias. Furthermore, there was significant statistical heterogeneity between most of the included studies. This variation was attributable to the difference in the demographic characteristics, surgical data, implant types, study designs, diagnostic workups, and follow-up periods. The random-effects model, subgroup analysis, and meta-regression analysis were performed to mitigate the potential impacts of this heterogeneity on the overall effect estimates. Subsequently, the published prospective and retrospective studies comprised incomplete data on history, presentation, histology, and management. The rarity of BIA-ALCL is challenging for healthcare providers with inadequate experience regarding diagnosis, reporting, and management of the disease.

## Conclusions

The current meta-analysis confirmed the association between breast implants and ALCL. The estimated risk of the disease ranged from 0 to 1 case per 1000 patients with breast implants. There was a relatively similar risk of BIA-ALCL among patients with aesthetic or reconstructive surgeries and patients with smooth or textured breast implants. Patients with a history of breast cancer were at higher risk of BIA-ALCL. This knowledge could help clinicians identify the current risk and the predictors of BIA-ALCL among breast implant patients. Identifying such evidence is critical for preventing the potential repercussions of BIA-ALCL on the future of implant-based breast surgeries.

## Supplementary Information

Below is the link to the electronic supplementary material.

Supplementary file1 (DOCX 28 KB)
